# Osmotic Adjustment and Antioxidant System Regulated by Nitrogen Deposition Improve Photosynthetic and Growth Performance and Alleviate Oxidative Damage in Dwarf Bamboo Under Drought Stress

**DOI:** 10.3389/fpls.2022.819071

**Published:** 2022-04-14

**Authors:** Shulan Wu, Jingqing Tian, Tingju Ren, Yanjie Wang

**Affiliations:** College of Life Science, Sichuan Normal University, Chengdu, China

**Keywords:** nitrogen deposition, drought stress, osmotic adjustment, antioxidative defense system, *Fargesia denudata*

## Abstract

Dwarf bamboo (*Fargesia denudata*) is a staple food for the endangered giant pandas and plays a critical role in the sub-alpine ecosystem. Characterized by shallow roots and expeditious growth, it is exceedingly susceptible to drought stress and nitrogen (N) deposition in the context of a changing global environment. However, a comprehensive picture about the interactive response mechanism of dwarf bamboo to the two factors, water regime and N deposition, is far from being given. Therefore, a completely randomized design with two factors of water regimes (well-watered and water-stressed) and N deposition levels (with and without N addition) of *F. denudata* was conducted. In view of the obtained results, drought stress had an adverse impact on *F*. *denudata*, showing that it destroyed ultrastructure integrity and induced oxidative damage and restricted water status in leaves and roots, as well as declined photosynthetic efficiency in leaves, especially in N non-deposition plants. Nevertheless, *F*. *denudata* significantly increased heat dissipation in leaves, regulated antioxidant enzymes activities, antioxidants contents, and osmoregulation substances concentrations in leaves and roots, as well as shifted biomass partitioning in response to drought stress. However, regardless of water availability, N deposition maintained better ultrastructure in leaves and roots, resulting in superior photosynthesis and growth of *F*. *denudata*. Additionally, although N deposition did not cause oxidative damage in well-watered plants, ameliorated the effects of drought stress on *F*. *denudata* through co-deploying heat dissipation in leaves, the antioxidant system in roots as well as osmotic adjustment in leaves and roots. Noticeably, the leaves and roots of *F*. *denudata* expressed quite distinct acclimation responses to drought resistance under N deposition.

## Introduction

With global climate change, the frequency and intensity of drought are increasing, causing plants to often suffer short-term or long-term drought stress. Drought, as one of the most serious abiotic stress factors, greatly restricts the growth, development, and natural regeneration of plants. Under drought, light energy absorption of plants exceeds the capacity for their light utilization due to the decline in photosynthesis (Medrano et al., 2002). The excess light energy that is not dissipated as fluorescence or heat will be inappropriately transferred to molecular oxygen, producing reactive oxygen species (ROS), such as superoxide anion (O_2_^-.^) and hydrogen peroxide (H_2_O_2_). Overproduction of ROS may cause oxidative damages to plants such as peroxidation of lipids, oxidation of proteins, and degradation of DNA and inhibition of enzyme activity (Sharma et al., 2012). Meanwhile, plants have evolved numerous defense mechanisms, such as thermal dissipation mediated by the xanthophyll cycle, antioxidant systems, and osmotic adjustment, to cooperate in preventing and alleviating oxidative damage (Gupta et al., 2015).

Thermal dissipation is measured as the non-photochemical quenching of chlorophyll fluorescence (NPQ), and the extent of NPQ is strongly regulated by the xanthophyll cycle. Many studies have shown that thermal dissipation is an important photo-protective mechanism in the prevention of photo-oxidative damage to plants (Zhang et al., 2017; Cheng et al., 2021). As another significant protective mechanism, the antioxidant defense system includes antioxidant enzymes such as superoxide dismutase (SOD), catalase (CAT), ascorbate peroxidase (APX), and glutathione reductase (GR) as well as antioxidants such as ascorbate (AsA) and glutathione (GSH), which play a significant role in scavenging and detoxifying ROS (Hasanuzzaman et al., 2020). Furthermore, osmotic adjustment is a key protective mechanism in plants under drought stress that is helpful to maintain cellular *osmotic homeostasis*, cell membrane integrity, and stabilization of enzymes/proteins as well as to detoxify ROS through the accumulation of compatible solutes such as soluble sugars, soluble proteins, and proline (Blum, 2017). Although these protective mechanisms have been well studied under drought, these results are still being debated and discrepancies possibly owing to plant species, plant organs, as well as duration and intensity of drought.

As one of the most important features of global change, atmospheric nitrogen (N) deposition has increased remarkably over the past century and is expected to continue increasing globally in the future (Yu et al., 2019). N deposition may be both a risk and an opportunity for plants and forest ecosystems, as it relies on the level of nitrogen input, plant species, N status of the forest ecosystems, and ecosystem types (Schulte-Uebbing and de Vries, 2018). The major impacts of N deposition appear in reducing terrestrial plant diversity, toxic effects on sensitive species, increasing susceptibility of plants to other stresses, and affecting plant physiological features as well as limiting plant growth (de Vries and Schulte-Uebbing, 2019). Therefore, understanding those responses and the mechanisms behind them is a major step to evaluate the N deposition impacts on plants.

In nature, multiple environmental stresses often occur simultaneously, especially under global environmental changes. Drought and N deposition, as two major factors in global climate changes, which severely impacts plant growth, structure and function, as well as forest ecosystems (Fusaro et al., 2017; Zheng et al., 2017). Some studies have reported that N deposition alleviates the negative effects of drought on plants by promoting photosynthesis and drought resistance (Zhong et al., 2019). However, other studies indicate that N deposition, promoting aboveground growth of plants, makes them more susceptible to drought (Shi et al., 2018; Wang et al., 2018). This controversy may result from species-specific properties, the intensity and duration of drought, and the level of N deposition. Therefore, the effect of the combination of drought and N deposition on plants remains not well understood.

As a rhizomatous, semi-woody and perennial evergreen plant, dwarf bamboos are extensively distributed in subtropical and tropical areas, and play an important role in preventing soil erosion and enhancing forest carbon sequestration (Tsuyama et al., 2012). *Fargesia denudata*, one of the dwarf bamboos and the most important dominant population in the lower layer of subalpine forests in China, is the staple food for the endangered giant pandas (Li et al., 2013). Therefore, maintaining a high productivity of *F. denudata* is very important for the giant panda’s survival and conservation. However, it is predicted that the distribution of dwarf bamboo will be narrowed due to global climate changes (Tuanmu et al., 2013; Yan et al., 2017). As a shallow-rooted plant, *F. denudata* is highly susceptible to drought, which seriously influences its productivity (Li et al., 2013). Moreover, N deposition will also have an impact on *F. denudata* due to its characteristics of rapid growth. Until now, much less attention has been paid to the response of *F. denudata* to N deposition as well as the combination of drought and N deposition.

Therefore, this study was executed to test this hypothesis that N deposition can alleviate the negative effects of drought on dwarf bamboo (*F. denudata*) through regulating thermal dissipation, antioxidant system, and osmotic adjustment to cooperatively protect against oxidative damage and improve photosynthetic and growth performance. In order to verify this hypothesis, plant growth parameters and water status as well as gas exchange and chlorophyll fluorescence parameters in leaves were determined. Simultaneously, the antioxidant system and osmotic solutes in leaves and roots were quantified. Furthermore, ultrastructure, ROS generation, and lipid peroxidation in leaves and roots were examined.

## Materials and Methods

### Plant Material and Experimental Design

The experiment was carried out at Maoxian Ecological Research Station, Chinese Academy of Sciences (103°53′58′′ E, 31°41′07′′ N, 1826 m a.s.l.) in southwestern China. *F. denudata* plants were collected from the nursery at Wanglang National Nature Reserve (103°55′ E, 32°49′ N, 2300 m a.s.l.). The healthy and uniform of *F. denudata* (2-year-old) were chosen to transplant into 50-L plastic pots filled with 35 kg of homogenized topsoil from the experimental site. One standard plant with 4–5 ramets was cultured in each pot. Afterwards, all plants were grown in a semi-controlled solar greenhouse with an ambient condition of 9–33°C and relative humidity of 40–85%, and watered every 3 days.

Seven months after transplantation, the experimental treatments, a completely randomized design with two factors of two water regimes and two N deposition levels，were applied. First, the two N deposition treatments were carried out: without N deposition [0 g N m^−2^ year^−1^ (−N): no addition of NH_4_NO_3_ solutions to each pot] and with N deposition [10 g N m^−2^ year^−1^ (+N), addition of 200 ml NH_4_NO_3_ solutions (6.18 mM N) to each pot weekly]. The amount of N addition was determined according to atmospheric N deposition rate in the study area (3.9 g N m^−2^ year^−1^; Lü and Tian, 2007). After 2 months of N deposition treatments, two water treatments, well-watered (80% relative soil water content, RSWC) and water-stressed (30% RSWC), were performed by withholding soil water for 30 days. The RSWC of each treatment was controlled through the weight method (Xu et al., 2009; Liu et al., 2017c). Each treatment had three replications with six standard plants per replication. To avoid systematic errors from differences in microclimate, all pots were randomly switched every week during the experiment. At the end of the experiment, various growth, physiological, and biochemical parameters were analyzed.

### Relative Water Content

Fresh leaves and roots were weighed immediately after collection to obtain the fresh weight (FW). After soaking leaves and roots in deionized water for 12 h at room temperature, this weight of turgid leaves and roots was considered as turgid weight (TW). Then the leaves and roots were dried at 70°C for 72 h and weighed the dry weight (DW). The relative water content (RWC) in leaves and roots were calculated as follows: RWC (%) = [(FW – DW)/(TW – DW)] × 100 (Galmés et al., 2007).

### Biomass Analysis

All plants were individually harvested and separated into leaves, stems, rhizomes, and roots. The plant parts were rinsed with distilled water, oven-dried at 70°C for 72 h, weighed, and recorded, respectively (Liu et al., 2014).

### Gas Exchange and Chlorophyll Fluorescence Parameters

The youngest fully expanded leaves at the same developmental stage were chosen to measure leaf gas exchange parameters including net photosynthetic rate (*P*_n_), stomatal conductance (*G*_s_), and intercellular CO_2_ concentration (*C*_i_) using a portable open-flow gas exchange system (LI-6400, LI-COR Inc., United States) during the late morning (9:00–11:00 am; local solar time). Leaves were placed in the LI-6400 chamber, which was adjusted to provide photon flux density (PPFD) of 800 μmol m^−2^ s^−1^, leaf temperature of 25°C, relative humidity of 60–70%, and CO_2_ concentration of 380 μmol·mol^−^ (Liu et al., 2017b,c).

The chlorophyll fluorescence in leaves (from plants used to measure gas exchange characteristics) was measured with a pulse-modulated fluorometer (FMS-2, Hansatech Instruments Ltd. Norfolk, United Kingdom). Under the conditions of activated light (800 μmol photons m^−2^ s^−1^), three parameters, light-adapted maximum (*F*_m_*′*), minimum (*F*_o_′), and steady-state fluorescence yield (*F*_s_), were determined simultaneously (Liu et al., 2017c). Then, the leaves were dark-adapted with leaf-clips for at least 30 min, and the minimum (*F*_o_) and maximum fluorescence yields (*F*_m_) were measured by detecting light (<0.05 μmol m^−2^ s^−1^) and a 0.8 s saturating pulse of white light (12,000 μmol m^−2^ s^−1^). Thereafter, the following parameters were recorded, including the maximum quantum efficiency of photosystem II (PSII; *F*_v_/*F*_m_) = (*F*_m_ – *F*_o_)/*F*_m_, photochemical quenching (*q*_p_) = (*F*_m_*′* – *F*_s_)/(*F*_m_*′* – *F*_o_*′* ), non-photochemical quenching (NPQ) = (*F*_m_ – *F*_m_*′*)/*F*_m_*′*, the quantum yield of PSII electron transport (Φ_PSII_) = (*F*_m_*′* – *F*_s_)/*F*_m_*′*, and open PSII reaction center excitation energy capture efficiency (*F*_v_*′*/*F*_m_*′*) = (*F*_m_*′* – *F*_o_*’*)/*F*_m_*′* (Xu and Zhou, 2006).

### Determination of Pigments

Pigments from xanthophyll cycle (V, violaxanthin; A, antheraxanthin; Z, zeaxanthin; L, lutein) of fresh leaves (0.3 g) were extracted in the dark by 80% acetone, filtered through a 0.45 μm membrane and analyzed with reversed-phase high-performance liquid chromatography (HPLC; Prominence UFLC, Shimadzu; Thayer and Björkman, 1990). A Spherisorb C18 column (5 μm, 250 mm × 4 mm) was used with a flow rate of 1.5 ml min^−1^. Elution was conducted with acetonitrile/methanol (75:25, v/v) and methanol/ethyl acetate (70:30, v/v) as the A and B mobile phase. The mobile phase gradient was used as follows: start with 100% A for 7 min, increase to 100% B within 2 min, and then maintained for 23 min. The column was re-equilibrated with 100% A for 5 min prior to the next injection. The 10 μl sample was injected, and the pigments were detected by absorption measurements at 445 nm. The de-epoxidation state (DEPS) of xanthophyll cycle was expressed as the percentage of (0.5A + Z)/(VAZ). Chlorophyll (Chl; Chl *a*, Chl *b*) was extracted in the dark from frozen leaf tissue (0.2 g) using 5 ml 100% acetone for 36 h at room temperature, and the absorbance was recorded at 662 nm and 645 nm, respectively (Xiong, 2003). The contents of Chl *a* and *b* were calculated using the following equations: Chl *a* = 11.75A662-2.35A645, Chl *b* = 18.61A645-3.96A662 (Şükran et al., 1998).

### Antioxidant Enzyme Activities and Antioxidants Analyses

Frozen leaves and roots (0.2 g) were extracted with 50 mM sodium phosphate buffer (SPB; pH 7.8) containing 0.2 mM EDTA, 2% (w/v) polyvinylpyrrolidone and 2 mM reduced ascorbate (AsA). The extract was centrifuged at 12,000 *g* for 20 min at 4°C, and the supernatant was instantly used for the antioxidant enzymes activities and soluble protein analyses.

Ascorbate peroxidase (APX; EC 1.11.1.11) activity was monitored by following the decrease of absorbance at 290 nm (*ε* = 2.8/(mM cm)) according to the modified method described in the previous paper (Nakano and Asada, 1981). The reaction mixture contained 25 mM SPB (pH 7.0), 0.1 mM EDTA, 5 mM AsA, 20 mM H_2_O_2_, and supernatant. Catalase (CAT; EC 1.11.1.6) activity was determined following the decomposition of H_2_O_2_ at 240 nm (*ε* = 39.4/(mM cm)) according to Cakmak and Marschner (1992). The reaction mixture contained 25 mM SPB (pH 7.0), 10 mM H_2_O_2_ and supernatant. Superoxide dismutase (SOD; EC 1.15.1.1) activity was estimated by the method of (Giannopolitis and Ries, 1977). The reactive mixture contained supernatant and nitroblue tetrazolium (NBT) solution (50 mM SPB (pH 7.8), 1.3 μM riboflavin, 63 μM NBT and 13 mM methionine). One unit of SOD activity was defined as the amount of enzyme required to cause a 50% inhibition in the rate of *p*-nitro blue tetrazolium chloride reduction at 560 nm.

Monodehydroascorbate reductase (MDHAR; EC 1.6.5.4) activity was estimated by detecting a decrease in absorbance at 340 nm (*ε* = 6.2/(mM cm) due to NADH oxidation according to Arrigoni et al. (1981). The reactive mixture contained 25 mM SPB (pH 7.8), 0.2 mM of EDTA, 0.1 mM AsA, 0.5 unit AsA oxidase, 4 mM NADH and supernatant. Dehydroascorbate reductase (DHAR; EC 1.8.5.1) activity was assayed by following the formation of AsA from ascorbate oxidized form dehydroascorbate (DHA) at 265 nm (*ε* = 14.6/(mM cm) as described by Dalton et al. (1986). The reactive mixture contained 25 mM SPB (pH 7.0), 0.1 mM EDTA, 8 mM DHA, 70 mM glutathione (reduced form, GSH) and supernatant. Glutathione reductase (GR; EC 1.6.4.2) activity was determined according to Madamanchi and Alscher (1991). The reaction mixture contained 25 mM SPB (pH 7.8), 0.2 mM EDTA, 2.4 mM NADPH, 10 mM oxidized glutathione (GSSG) and supernatant. The decrease in the absorbance caused by NADPH oxidation was determined at 340 nm (*ε* = 6.2/(mM cm)).

The levels of total ascorbate (AsA + DHA), AsA, and DHA were measured according to the modified method of Law et al. (1983). Frozen leaves and roots (0.2 g) were homogenized in 2 ml of 5% (w/v) ice-cold TCA, and centrifuged at 15,000 *g* for 15 min at 4°C. For AsA + DHA measurement, the reaction mixture contained 0.2 ml of the supernatant, 0.5 ml of 150 mM SPB with 5 mM EDTA (pH 7.4), and 0.1 ml of 10 mM dithiothreitol (DTT). After incubation for 10 min at room temperature, 0.05 ml of 0.5%N-ethylmaleimide was added to remove excess DTT. For AsA determination, the reaction mixture included 0.2 ml of the supernatant, 0.5 ml of 150 mM SPB with 5 mM EDTA (pH 7.4), and 0.2 ml of deionized H_2_O. Color was developed in both reaction mixtures after the addition of the following reagents: 0.4 ml of 10%TCA, 0.4 ml of 44% orthophosphoric acid, 0.4 ml of 4% 2,2′-bipyridyl, and 0.2 ml of 3% FeCl_3_. The mixtures were then incubated at 40°C for 40 min and quantified at 525 nm. DHA was estimated from the difference between AsA + DHA and AsA. A standard curve was built based on AsA.

The concentrations of total glutathione (GSH + GSSG), GSH, and GSSG were determined by the 5,5′-dithiobisnitrobenzoic acid (DTNB)-GR recycling procedure following by the modified method of Zhou et al. (2007). Frozen leaves and roots (0.2 g) were extracted with 6% (w/v) ice-cold metaphosphoric acid and centrifuged at 12,000 *g* for 20 min at 4°C. In the case of GSH + GSSG assay, the reactive mixture included 1.6 ml of 100 mM SPB (pH 7.5), 0.1 ml of 0.6 mM 5,5′-dithiobis-2-nitrobenzoic acid (DTNB), 0.1 ml of 0.2 mM NADPH, 0.1 ml of 50 U/ml GR, and 0.1 ml of the supernatant and quantified at 412 nm. GSSG was determined in the same way except that 0.1 ml of the supernatant was pretreated with 0.03 ml of 2-vinylpyridine at 25°C for 1 h. GSH was determined by subtraction of GSSG from the GSH + GSSG. A standard curve was used based on GSSG.

### Measurement of Osmotic Adjustment Substances

Dry leaves and roots (0.1 g) were extracted with 6 ml of 80% ethanol at 80°C for 30 min, and centrifuged at 3,000 *g* for 10 min, and then the supernatant was collected. This above step was repeated for three times. The combined supernatant was used for analysis of soluble sugar by anthrone method (Zhang and Qu, 2003). Soluble protein was measured following Bradford’s method (Bradford, 1976). The supernatant was determined with Bradford G-250 reagent at 595 nm using bovine serum albumin (BSA) as a calibration standard.

Proline content was determined according to the modified method of Bates et al. (1973). Fresh leaves and roots (0.1 g) were extracted with 3% sulfosalicylic acid and filtered through filter paper. Glacial acetic acid and ninhydrin (1.25 g ninhydrin, 30 ml of glacial acetic acid, 20 ml of 6 M H_3_PO_4_) solutions were added to 2 ml of the extract. The reactive mixture was heated at 100°C for 1 h and quickly cooled on ice, and then 4 ml of toluene was added. Finally, the content of proline (in the upper hydrophobic phase) was determined at 520 nm using L-proline as a standard.

### Measurement of ROS and Lipid Peroxidation

The histochemical staining of superoxide anion (O_2_^−^) and hydrogen peroxide (H_2_O_2_) in leaves were performed by the modified method according Thordal-Christensen et al. (1997) to. In the case of O_2_^−^, fresh leaves were vacuum infiltrated directly with 0.1 mg mL^−1^ NBT in 25 mM K-HEPES buffer (pH 7.8), then incubated at 25°C in the dark for 2 h. In the case of H_2_O_2_, fresh leaves were vacuum infiltrated immediately with 1 mg mL^−1^ DAB in 50 mM Tris-acetate (pH 3.8), then incubated at 25°C in dark for 24 h. In both cases, leaves were rinsed in 80% (v/v) ethanol for 10 min at 70°C, mounted in lactic acid/phenol/water (1:1:1; v/v), and photographed.

H_2_O_2_ concentration was determined by following the method of Bian and Jiang (2009). Fresh leaves and roots (0.2 g) were homogenized in ice-cold acetone and centrifuged at 3,000 *g* for 10 min at 4°C. The reactive mixture included 1 ml of the supernatant, 0.2 ml of ammonia and 0.1 ml of 20% (v/v) titanium tetrachloride (20% TiCl_4_ in HCl), and then centrifuged at 3,000 *g* for 10 min. The obtained precipitate was washed five times with ice-cold acetone (to remove the pigments) and then centrifuged at 10,000 *g* for 5 min. Finally, the pellet was solubilized with 3 ml of 1 M H_2_SO_4_ and measured at 410 nm. The H_2_O_2_ content was calculated using a standard curve.

The production rate of O_2_^−^ was estimated according to the modified method of Bian and Jiang (2009). Fresh leaves and roots (0.2 g) were homogenized with 2 ml of 65 mM ice-cold SPB (pH 7.8) and then centrifuged at 5,000 *g* for 10 min at 4°C. The reactive mixture, including l ml of the supernatant, 0.9 ml of 65 mM SPB (pH 7.8) and 0.1 ml of 10 mM hydroxylamine hydrochloride, was incubated for 20 min at 25°C. The above solution (1 ml) was mixed with 1 ml of 17 mM 4-aminobenzenesulfonic acid and 1 ml of 7 mM α-naphthylamine, and then kept for 20 min at 25°C. Finally, the absorbance of the supernatant was measured at 530 nm.

Lipid peroxidation was determined by measuring the malondialdehyde (MDA) content following by the thiobarbituric acid (TBA) test Bian and Jiang (2009). 0.2 g of frozen leaves and roots were homogenized with 2 ml of 50 mM ice-cold SPB (pH 7.8) and then centrifuged at 12,000 *g* for 20 min at 4°C. The above supernatant (1 ml) was mixed with 3 ml of 20% (w/v) TCA solution containing 2% (w/v) TBA at 95°C for 30 min, and then quickly cooled on ice. After centrifugation at 15,000 *g* for 10 min, the MDA content was recorded at 532 nm and 600 nm.

### Ultrastructural Observations

The small sections (1–2 mm in length) of fresh leaves and roots were instantly fixed in 3% glutaraldehyde in 0.1 M SPB (pH 7.2) for 8 h at 4°C, then fixed in 1% osmium tetroxide for 2 h and immersed in 0.1 M phosphate buffer for 2 h. The tissue sections were dehydrated through a graded ethanol series (50, 60,70, 80, 90, 100%, 20 min each step) and embedded in Epon 812. Ultrathin sections (80 nm) were cut using an ultramicrotome (Reichert-Jung Ultracut E), stained with uranylacetate and lead citrate and mounted onto copper grids for viewing in the H-600IV TEM (Hitachi, Tokyo, Japan; Liu et al., 2017a).

### Statistical Analysis

All obtained data were analyzed by one-way analysis of variance (ANOVA), and Duncan’s test was used to compare the means on a value of *p* < 0.05 using the statistical software (SPSS Inc., Chicago, United States).

## Results

### Relative Water Content

**Figure 1:**
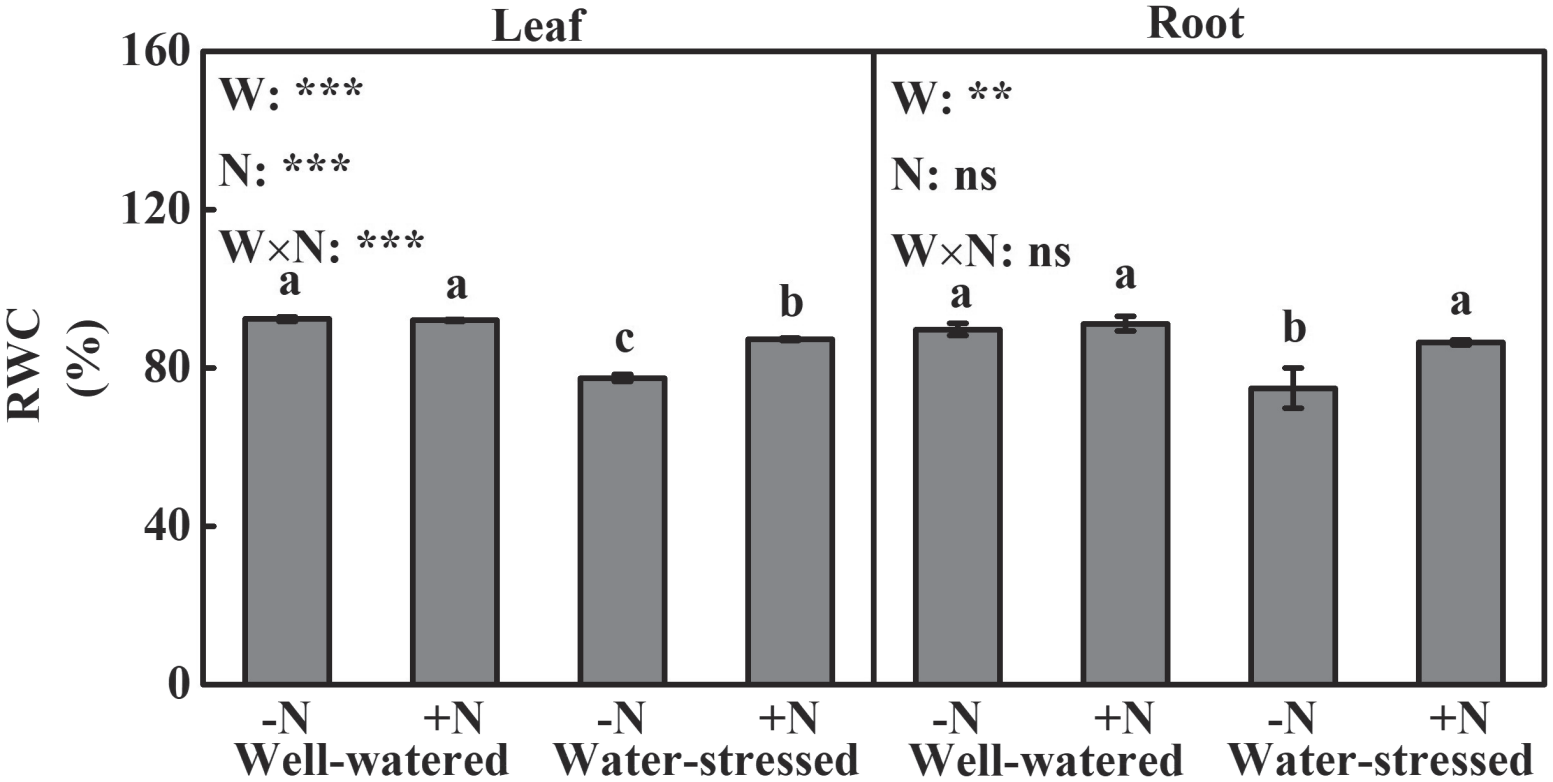
Leaf relative water content of *Fargesia denudata* Yi plants for N non-deposition (−N) and N deposition (+N) treatments with and without drought stress. *F*_W_, water effect; *F*_N_, N deposition effect; and *F*_W_ × *F*_N_, interactive effect of water and N deposition. Values with different letters are significantly different at *p* < 0.05. Vertical bars show ± S.E. Significant levels: ^***^p < 0.001; ^**^p < 0.01; ns (non-significant) *p* > 0.05.

We found significant effects of drought stress, N deposition and their interaction on RWC in leaf. In comparison with well-watered plants, drought stress significantly decreased RWC by 5.23 and 16.12%, respectively, in N deposition and N non-deposition plants. Although N deposition did not significantly change RWC in well-watered plants, whereas it markedly increased RWC (12.67%) in water-stressed plants compared with N non-deposition (Figure 1A). Compared to well-watered plants, RWC in roots was significantly decreased (16.56%) in N non-deposition plants by drought stress. Similar to the results in leaves, N deposition only significantly increased RWC by 15.37% in roots in water-stressed plants compared with N non-deposition (Figure 1B).

### Plant Biomass

For all of the biomass parameters, a significant effect of drought stress on stem biomass, and of N deposition on leaf biomass, stem biomass and total biomass were detected, but all biomass indexes were not affected by the interaction of N deposition and drought-stress. Regardless of N deposition, these biomass parameters in water-stressed groups were lower compared to well-watered groups, expect for root biomass. Interestingly, in both watering conditions, N deposition increased all biomass parameters Moreover, N deposition notably increased leaf biomass, stem biomass, and total biomass compared to N non-deposition (Table 1).

**Table 1:** Biomass of *Fargesia denudata* plants under N non-deposition (−N) and N deposition (+N) treatments with and without drought stress.

Traits	Well-watered		Water-stressed		Effects		
	−N	+N	−N	+N	*F*_W_	*F*_N_	*F*_W_ × *F*_N_
Leaf biomass	19.25 ± 3.58b	32.86 ± 2.11a	16.14 ± 1.37b	32.08 ± 2.09a	0.64^ns^	37.08^***^	0.23^ns^
Stem biomass	34.78 ± 3.32c	55.39 ± 4.77a	28.89 ± 0.61c	45.41 ± 0.35b	7.35^*^	40.30^***^	0.49^ns^
Root biomass	21.12 ± 4.66b	33.71 ± 7.25ab	30.21 ± 2.92ab	39.90 ± 4.95a	2.18^ns^	4.63^ns^	0.08^ns^
Rhizome biomass	59.45 ± 7.23ab	71.55 ± 8.94a	48.98 ± 2.01b	63.79 ± 2.06ab	2.36^ns^	5.15^ns^	0.05^ns^
Total biomass	134.59 ± 11.07b	193.51 ± 11.65a	124.23 ± 2.96b	181.18 ± 7.19a	1.62^ns^	42.11^***^	0.01^ns^

F_W_, water effect; F_N_, phosphorus effect; and F_W_ × F_N_, interactive effect of water and N deposition. All the tested biomasses were expressed as g plant^−1^ DW. Means followed by the same letter are not significantly different at p < 0.05 according to Duncan’s test. Data are means ± S.E. Significant levels: ^***^p < 0.001; ^*^p < 0.05; ns (non-significant) p > 0.05.

### Gas Exchange and Chlorophyll Fluorescence Parameters

**Figure 2:**
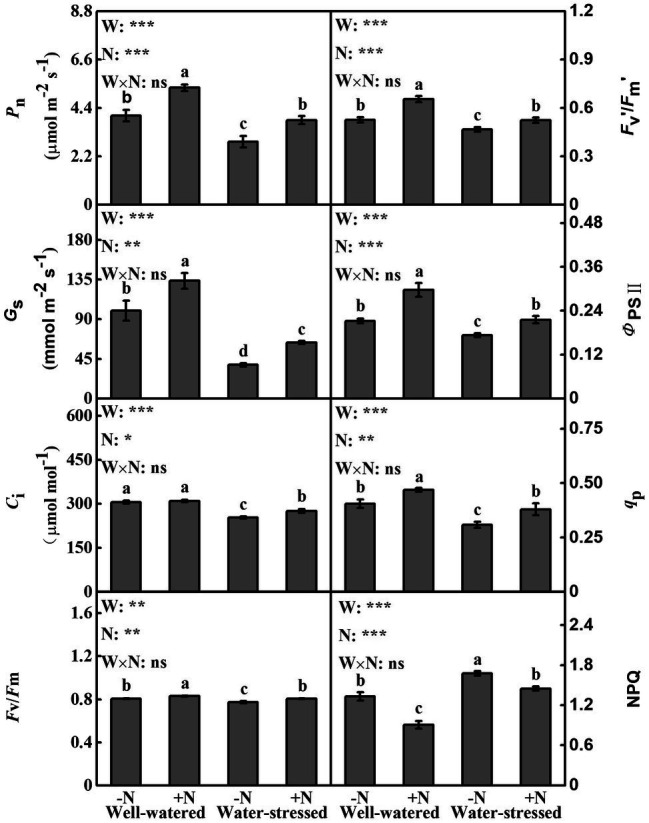
Gas exchange and chlorophyll fluorescence parameters of *Fargesia denudata* leaves for N non-deposition (−N) and N deposition (+N) treatments with and without drought stress. *F*_W_, water effect; *F*_N_, N deposition effect; and *F*_W_ × *F*_N_, interactive effect of water and N deposition. Values with different letters are significantly different at *p* < 0.05. Vertical bars show ± S.E. Significant levels: ^***^p < 0.001; ^**^p < 0.01; ^*^p < 0.05; ns (non-significant) *p* > 0.05.

Drought stress and N deposition had a significant effect individually on all gas exchange and chlorophyll fluorescence parameters; however, no significant change was found in its interactive effects. Regardless of N deposition, compared with well-watered plants, drought stress caused a significant decline in *P*_n_, *G*_s_, *C*_i_, *F*_v_/*F*_m_, *F*_v_*’*/*F*_m_*′*, *q*_P_ and Φ_PSII_, but a significant increase in NPQ. However, the opposite trend was apparent in N deposition regardless of water availability. Concurrently, although N deposition had little effect on *C*_i_ in well-watered plants, it significantly increased *C*_i_ by 8.54% in water-stressed plants compared to N non-deposition (Figure 2).

### Pigments

Chl*a*, Chl*b*, and L in fresh leaves were significantly affected by drought stress and N deposition. However, the interaction between drought stress and N deposition had not affected all pigments. Compared with well-watered plants, drought stress significantly dampened Chl*a* and Chl*b*, but increased L and DEPS regardless of N deposition. In contrast, in both watering conditions, compared with N non-deposition plants, N deposition increased dramatically Chl*a* and Chl*b*, but decreased L and DEPS. (Table 2).

**Table 2:** Pigments content of *Fargesia denudata* leaves for N non-deposition (−N) and N deposition (+N) treatments with and without drought stress.

Traits	Well-watered		Water-stressed		Effects		
	−N	+N	−N	+N	*F*_W_	*F*_N_	*F*_W_ × *F*_N_
Chl*a*	4.05 ± 0.25b	7.42 ± 0.47a	2.59 ± 0.07c	4.81 ± 0.22b	49.29^***^	92.56^***^	3.93^ns^
Chl*b*	1.19 ± 0.07b	2.09 ± 0.12a	0.76 ± 0.04c	1.46 ± 0.08b	42.08^***^	94.45^***^	1.62^ns^
L	193.16 ± 5.32b	182.71 ± 1.95b	211.38 ± 6.16a	195.75 ± 1.97b	13.22^**^	9.19^*^	0.36^ns^
VAZ	85.55 ± 1.49a	84.69 ± 3.38a	85.04 ± 2.85a	85.86 ± 0.96a	0.02^ns^	<0.01^ns^	0.13^ns^
DEPS	20.70 ± 0.18a	19.16 ± 0.69a	21.53 ± 1.42a	20.00 ± 0.21a	1.09^ns^	3.68^ns^	<0.01^ns^

F_W_, water effect; F_N_, phosphorus effect; and F_W_ × F_N_, interactive effect of water and N deposition. Lutein (L), violaxanthin (V), antheraxanthin (A), zeaxanthin (Z), and xanthophyll cycle pool (VAZ) were expressed as mmol mol^−1^ Chla + b; the de-epoxidation state of xanthophyll cycle pool (DEPS, %) is calculated as the percentage of (0.5A + Z)/VAZ; chlorophyll a, b (Chla, b) were expressed as mg g^−1^ FW. Means followed by the same letter are not significantly different at p < 0.05 according to Duncan’s test. Data are means ± S.E. Significant levels: ^***^p < 0.001; ^**^p < 0.01; ^*^p < 0.05; ns (non-significant) p > 0.05.

### Antioxidant Enzymes and Antioxidants

**Figure 3:**
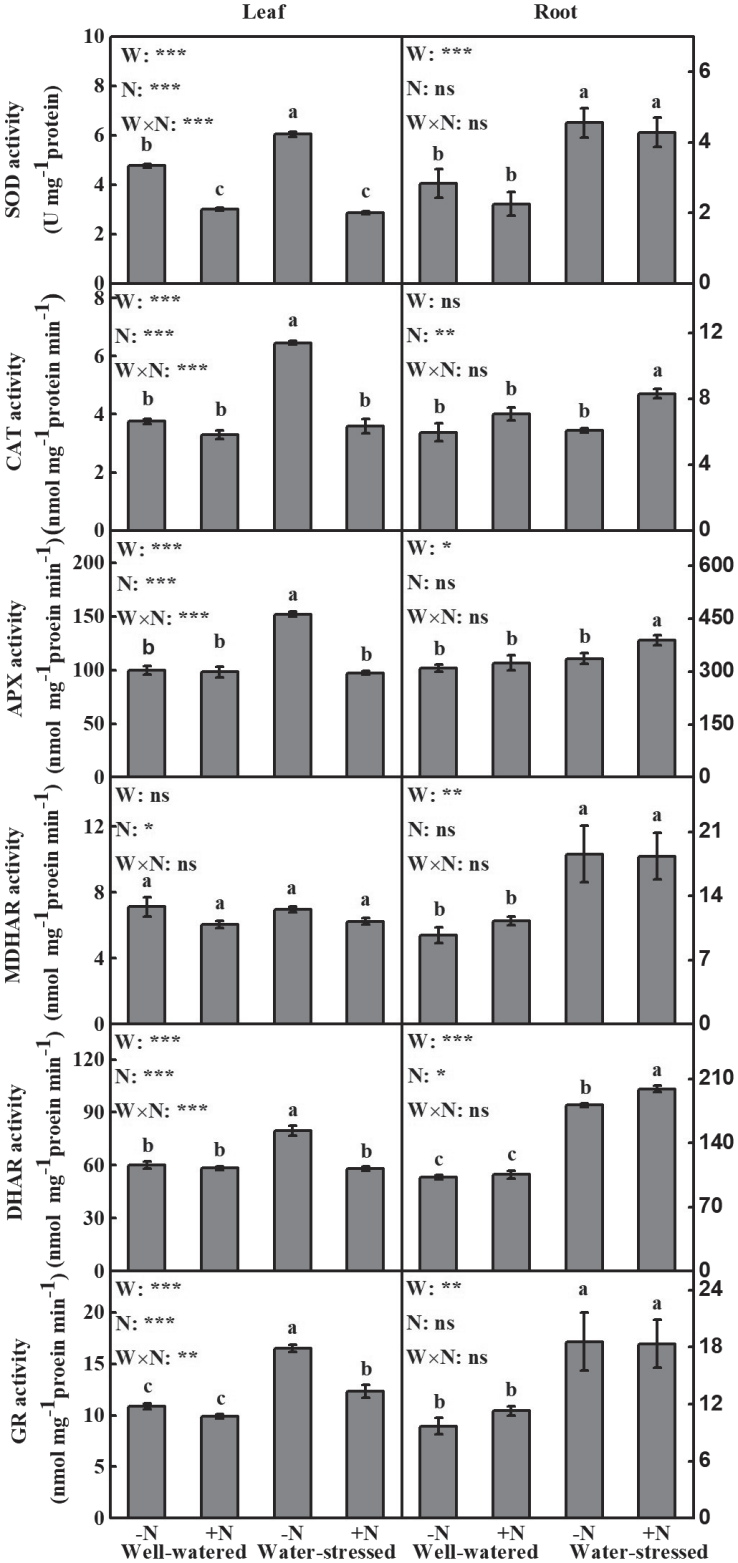
Antioxidant enzymes of *Fargesia denudata* leaves and roots for N non-deposition (−N) and N deposition (+N) treatments with and without drought stress. *F*_W_, water effect; *F*_N_, N deposition effect; and *F*_W_ × *F*_N_, interactive effect of water and N deposition. SOD, superoxide dismutase; CAT, catalase; APX, ascorbate peroxidase; MDHAR, monodehydroascorbate reductase; DHAR, dehydroascorbate reductase; GR, glutathione reductase. Values with different letters are significantly different at *p* < 0.05. Vertical bars show ± S.E. Significant levels: ^***^p < 0.001; ^**^p < 0.01; ^*^p < 0.05; ns (non-significant) *p* > 0.05.

The content of antioxidants in leaves and roots (except MDHAR in leaves and CAT in roots), AsA + DHA, AsA, AsA/DHA, and GSH in leaves as well as AsA in roots were in general affected by drought stress. N deposition significantly influenced all antioxidant contents, AsA, AsA/DHA in leaves as well as CAT, DHAR, and AsA + DHA in roots. Furthermore, the interaction of the two factors had significant impact on all antioxidants (except MDHAR), AsA, AsA/DHA, GSH, and GSH + GSSG in leaves, while only AsA in roots (Figure 3, Table 3).

**Table 3:** Antioxidants contents of *Fargesia denudata* leaves and roots for N non-deposition (−N) and N deposition (+N) treatments with and without drought stress.

Organs/Traits		Well-watered		Water-stressed		Effects		
		−N	+N	−N	+N	*F*_W_	*F*_N_	*F*_W_ × *F*_N_
Leaf	AsA + DHA	4.95 ± 0.27b	4.54 ± 0.28b	6.32 ± 0.44a	5.31 ± 0.20ab	11.89^**^	5.22^ns^	0.94^ns^
	AsA	1.51 ± 0.10bc	1.28 ± 0.13c	3.38 ± 0.17a	1.83 ± 0.11b	86.66^***^	46.88^***^	26.06^***^
	AsA/DHA	0.45 ± 0.06bc	0.39 ± 0.03c	1.10 ± 0.03a	0.52 ± 0.02b	123.31^***^	80.00^***^	54.44^***^
	GSH + GSSG	4.24 ± 0.29ab	3.56 ± 0.08b	5.03 ± 0.47a	4.13 ± 0.27ab	4.86^ns^	6.53^*^	0.14^ns^
	GSH	2.94 ± 0.19b	2.55 ± 0.14b	3.80 ± 0.32a	2.93 ± 0.22b	7.30^ns^	7.51^*^	1.07^*^
	GSH/GSSG	2.42 ± 0.41a	2.57 ± 0.31a	3.34 ± 0.72a	2.48 ± 0.24a	0.82^ns^	0.59^ns^	1.20^ns^
Root	AsA + DHA	1.27 ± 0.11a	0.78 ± 0.14b	1.01 ± 0.08ab	0.94 ± 0.09ab	0.20^ns^	7.00^*^	4.15^ns^
	AsA	0.82 ± 0.02a	0.42 ± 0.01c	0.65 ± 0.02b	0.68 ± 0.02b	8.10^*^	121.00^***^	165.02^***^
	AsA/DHA	1.84 ± 0.88a	1.53 ± 0.48a	2.08 ± 0.50a	3.53 ± 1.15a	1.94^ns^	0.51^ns^	1.19^ns^
	GSH + GSSG	0.14 ± 0.02a	0.16 ± 0.03a	0.12 ± 0.01a	0.17 ± 0.02a	0.15^ns^	2.11^ns^	0.64^ns^
	GSH	0.06 ± 0.01a	0.05 ± 0.01a	0.04 ± 0.01a	0.06 ± 0.01a	0.37^ns^	0.48^ns^	2.77^ns^
	GSH/GSSG	0.77 ± 0.08a	0.47 ± 0.10a	0.47 ± 0.11a	0.60 ± 0.06a	0.84^ns^	0.96^ns^	5.68^ns^

F_W_, water effect; F_N_, N deposition effect; and F_W_ × F_N_, interactive effect of water and N deposition. The reduced (AsA) and the oxidized (DHA) form of ascorbate as well as the reduced (GSH) and the oxidized (GSSG) form of glutathione were expressed as μmol g^−1^. Means followed by the same letter are not significantly different at p < 0.05 according to Duncan’s test. Data are means ± S.E. Significant levels: ^***^p < 0.001; ^**^p < 0.01; ^*^p < 0.05; ns (non-significant) p > 0.05..

Compared to well-watered plants, drought stress significantly increased most antioxidant enzymes activities in leaves in N non-deposition plants, showing significantly higher activities of SOD (26.73%), CAT (72.2%), APX (52.1%), DHAR (32.61%), and GR (51.56%), while only GR activity significantly increased in N deposition plants (Figure 3). Meanwhile, regardless of N deposition, the contents of AsA + DHA, AsA, AsA/DHA, GSH + GSSG, and GSH were higher in water-stressed plants than well-watered plants (Table 3). Additionally, in comparison with N non-deposition plants, N deposition only significantly decreased SOD activity in well-watered plants; moreover, it reduced the activities of SOD, CAT, APX, DHAR, GR, the contents of AsA + DHA, AsA, and GSH as well as AsA/DHA ratio in water-stressed plants.

Compared to well-watered plants, almost all antioxidant enzymes activities (SOD, MDHAR, DHAR, GR) were significantly increased in roots, but AsA content was significantly decreased in N non-deposition plants by drought stress. Meanwhile, drought stress significantly increased all antioxidant enzymes activities as well as AsA content in N deposition plants. In addition, only the contents of AsA + DHA and AsA were significantly decreased in well-watered plants by N deposition (Figure 3; Table 3). However, in water-stressed conditions, N deposition significantly increased the activities of CAT, APX, and DHAR by 36.86, 15.24, and 9.75%, respectively, compared with their N non-deposition counterparts (Figures 3H,I,K).

### Osmotic Adjustment

**Figure 4:**
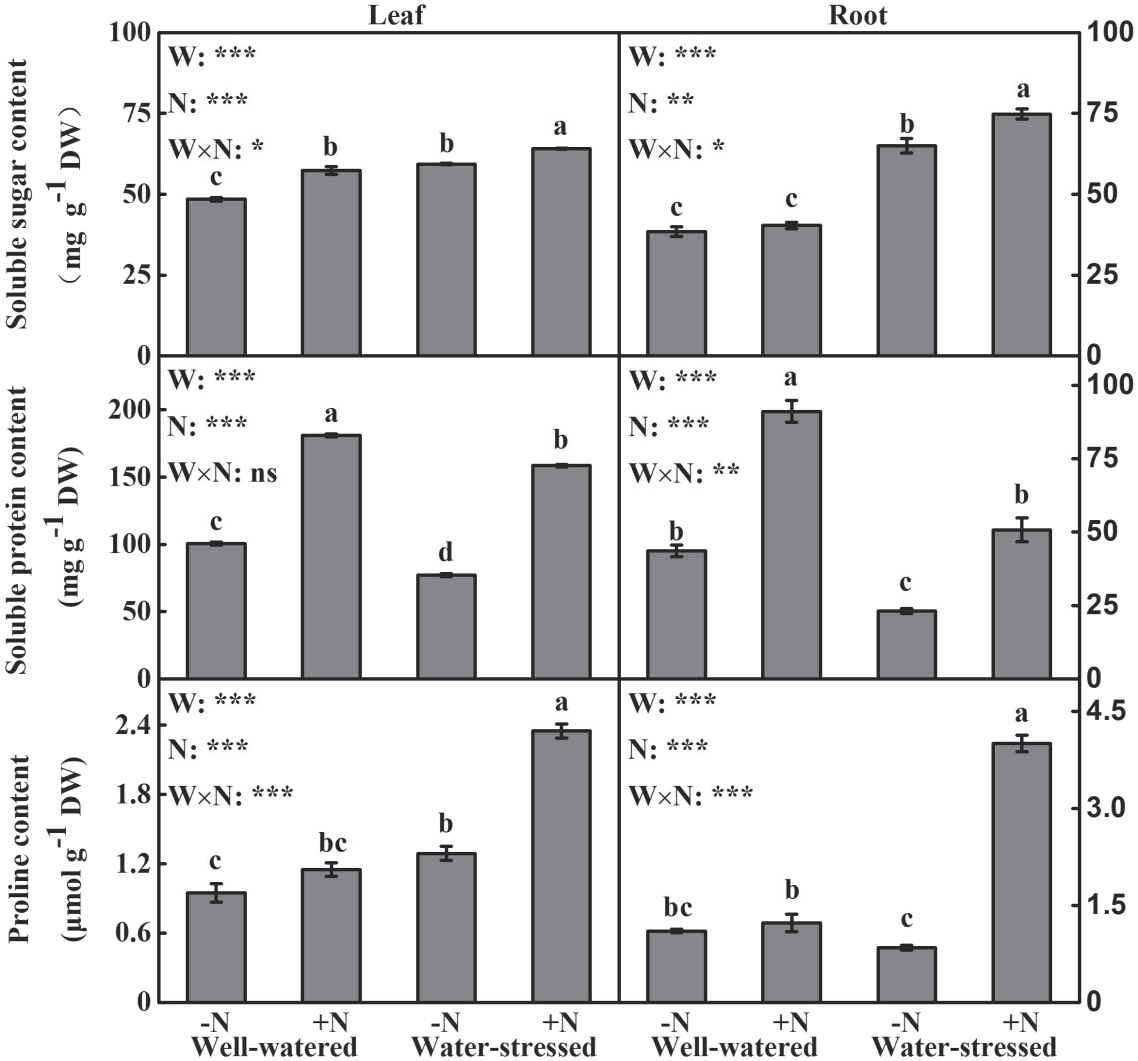
Osmotic adjustment substances of *Fargesia denudata* leaves and roots for N non-deposition (−N) and N deposition (+N) treatments with and without drought stress. *F*_W_, water effect; *F*_N_, N deposition effect; and *F*_W_ × *F*_N_, interactive effect of water and N deposition. Values with different letters are significantly different at *p* < 0.05. Vertical bars show ± S.E. Significant levels: ^***^p < 0.001; ^**^p < 0.01; ^*^p < 0.05; ns (non-significant) *p* > 0.05.

Except that soluble protein in leaves was not significantly affected by interactive effect of the two factors, other osmotic adjustment substances in leaves and roots were significantly influenced by the two factors individually and interactively (Figure 4). Regardless of N deposition, drought stress significantly increased soluble sugar content in leaves and roots, while it significantly decreased soluble protein content. Concomitantly, drought stress significantly increased proline content in leaves irrespective of N deposition, while it only significantly increased proline content in roots in N deposition plants. In addition, soluble sugar content in leaves as well as soluble protein content in leaves and roots under well-watered conditions with N deposition were greater than those in N non-deposition plants. Moreover, in water-stressed conditions, the contents of three osmoregulation substances in leaves and roots were significantly enhanced by N deposition (Figure 4).

### ROS and MDA

**Figure 5:**
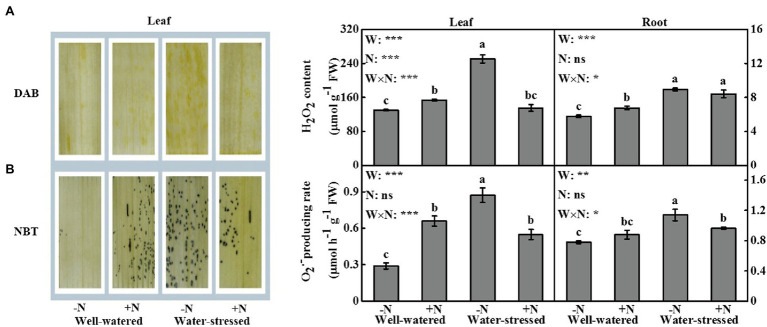
*In situ* detection of reactive oxygen species (ROS) in leaves, and quantitative measurements of ROS in leaves and roots of *Fargesia denudata* for N non-deposition (−N) and N deposition (+N) treatments with and without drought stress. **(A)** hydrogen peroxide (H_2_O_2_) accumulation, **(B)** producing rate of superoxide anion ( $O_{2}^{\cdot -}$ ). *F*_W_, water effect; *F*_N_, N deposition effect; and *F*_W_ × *F*_N_, interactive effect of water and N deposition. Values with different letters are significantly different at *p* < 0.05. Vertical bars show ± S.E. Significant levels: ^***^p < 0.001; ^**^p < 0.01; ^*^p < 0.05; ns (non-significant) *p* > 0.05.

**Figure 6:**
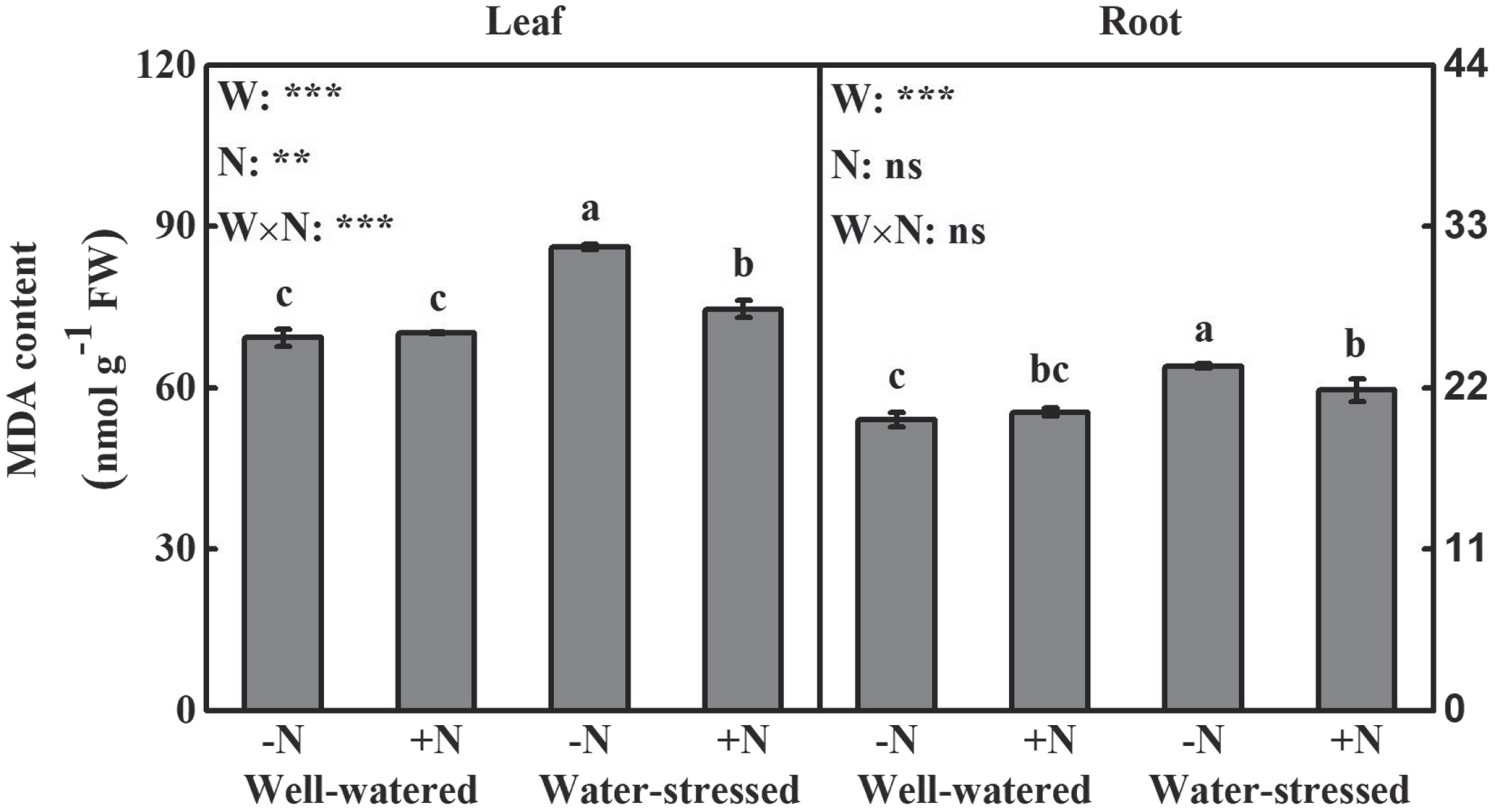
Lipid peroxidation (MDA) of *Fargesia denudata* leaves and roots for N non-deposition (−N) and N deposition (+N) treatments with and without drought stress. *F*_W_, water effect; *F*_N_, N deposition effect; and *F*_W_ × *F*_N_, interactive effect of water and N deposition. Values with different letters are significantly different at *p* < 0.05. Vertical bars show ± S.E. Significant levels: ^***^p < 0.001; ^**^p < 0.01; ns (non-significant) *p* > 0.05.

Drought stress significantly affected ROS accumulation and MDA content in leaves and roots. N deposition only significantly influenced the contents of H_2_O_2_ and MDA in leaves (Figures 5, 6). The interactive effect of the two factors was significant on the levels of ROS and MDA in leaves; similar findings were detected in roots (except MDA content in roots; Figures 5, 6).

According to the results of DAB and NBT staining in leaves (Figures 5A,B), the accumulation of H_2_O_2_ and $O_{2}^{\cdot -}$ was the highest in the treatment of drought stress with N non-deposition, followed by that of drought stress with N deposition and sufficient moisture with N deposition, while the accumulation of ROS was the least in the treatment of sufficient moisture with N non-deposition.

In leaves and roots, H_2_O_2_ content, the production rate of $O_{2}^{\cdot -}$ ^,^ and MDA content were significantly higher in water-stressed plants than in well-watered plants under N non-deposition conditions. Drought stress significantly increased H_2_O_2_ content in roots as well as MDA content in leaves in N deposition plants when compared with well-watered counterparts. Additionally, in comparison with well-watered plants, N deposition caused higher levels of ROS in leaves and roots, while no significant change in MDA content in well-watered plants. However, it significantly reduced ROS accumulation and MDA content in leaves, but significantly decreased the production rate of $O_{2}^{\cdot -}$ and MDA content in roots in water-stressed plants.

### Ultrastructure

**Figure 7:**
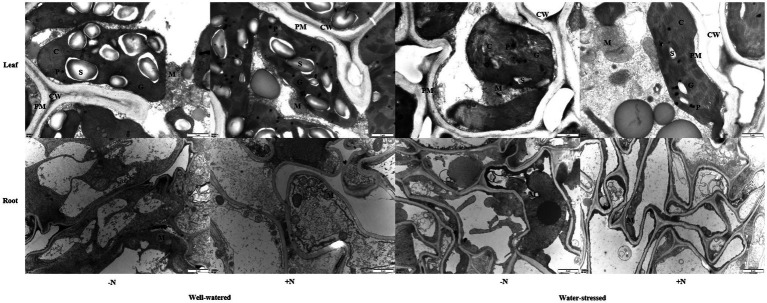
Ultrastructural observations of leaves and roots of *Fargesia denudata* plants for N non-deposition (−N) and N deposition (+N) treatments with and without drought stress. The bars shown are 1 μm of leaf and 2 μm of root. C, chloroplast; CW, cell wall; G, granum; M, mitochondria; P, plastoglobulus; PM, plasma membrane; S, starch granule.

Drought stress leads to worse ultrastructure in leaves and roots of *F*. *denudata* than those in well-watered plants, particularly in N non-deposition conditions, showing a certain degree of plasmolysis, swollen and deformed chloroplasts, broken and loosely arranged basal lamellae, damaged chloroplast membrane, and lower starch granules. Meanwhile, the integrity of the roots’ mitochondrial membrane was obviously damaged, mitochondrial internal materials were seriously degraded, and mitochondrial crista had partially disappeared. However, when compared with N non-deposition plants, N deposition increased the number of basal lamellae and the degree of stacking in chloroplast in leaves and the number of mitochondria in roots under well-watered conditions. Moreover, N deposition alleviated the damage to the ultrastructure in leaves and roots under drought stress; the swelling and deformation were eased, the loss of normal shape and arrangement of basal lamellae had declined, and the membrane was relatively integrity in chloroplasts. Additionally, the membrane integrity was not obviously damaged, and the internal degradation and crista disappearance were lessened in the mitochondria of roots (Figure 7).

## Discussion

### Effects of Drought Stress and N Deposition on Relative Water Content and Biomass

Being an effective measurement method of the stress intensity on plants, RWC is a physiological indicator of cellular moisture stress and drought tolerance (Singh and Singh, 2003; Rosales-Serna et al., 2004; Shivakrishna et al., 2018). Previous studies found that RWC in leaves and roots decrease significantly as the degree of drought stress deepens (Liu et al., 2017c; Han et al., 2019). Similarly, our study showed that *F. denudata* generally displayed sharp decreases in RWC of leaves and roots under drought stress irrespective of N deposition (Figure 1), suggesting that drought stress could cause severe water loss in leaf and root cells. In addition, we found no significant changes in RWC of leaves and roots caused by N deposition in well-watered conditions, as was similar to the results in *Phyllostachys edulis* under well-watered plants (Wu et al., 2018). However, N deposition obviously increased RWC of leaves and roots in water-stressed plants (Figure 1). Accordingly, our study suggested that the extent of drought-induced water loss in *F*. *denudata* was ameliorated by means of N deposition, which might be related to stronger osmotic adjustment ability to absorb water (Loutfy et al., 2012; Zhong et al., 2017). Similarly, nitrogen application also increased RWC of leaves in *Coffea canephora* Pierre, *Eucalyptus grandis*, and *Sophora davidii* seedlings subject to water deficit (DaMatta et al., 2002; Graciano et al., 2005; Wu et al., 2008).

During plant growth and development, water and nutrients, especially N, are strong driving factors in growth and productivity and have significant interactive effects on plants (Yin et al., 2009, 2012). It has been concluded that N deposition has a certain ecologically compensative effect on plants under drought stress (Song et al., 2019; Wang et al., 2019). In this work, drought stress promoted root growth, whereas it partly restricted other parts’ growth in *F*. *denudata*, regardless of N deposition (Table 1). An explanation for this is that when *F*. *denudata* face water shortage, biomass partitioning shifted in order to reduce water evaporation and concurrently stimulate water absorption from soil, resulting in raised roots at the expense of other parts (Studer et al., 2017), thereby enabling *F*. *denudata* to survive in drought stress. Similar findings were reported in the previous studies (Liu et al., 2014; Studer et al., 2017). Additionally, a certain amount of nitrogen application also promoted other plants’ growth under different watering conditions (Du et al., 2017; Xiong et al., 2018; Wang et al., 2019). Our study likewise revealed that N deposition boosted the growth of *F*. *denudata* under well-watered and water-stressed conditions (Table 1), which may be attributed to the improvement of water status and photosynthetic performance in water-stressed plants by N deposition (Figures 1, 2). Shi et al. (2017) considered that N-use efficiency and transcription of related-genes encoding N metabolism enzymes were elevated by nitrogen application under drought stress, thus positively contributing to drought resistance and growth of *Catalpa bungei*.

### Effects of Drought Stress and N Deposition on Photosynthetic Fluorescence Characteristics

Photosynthesis is a crucial metabolic process for plants to grow with solar energy; however, it is particularly sensitive to environmental conditions (Chaves et al., 2009). Recent studies have demonstrated that water deficit severely restricted the photosynthetic efficiency of plants (Wu et al., 2018; Han et al., 2019). In this work, a significant lowering in Chl contents as well as gas exchange parameters of *F*. *denudata* was observed after drought stress regardless of N deposition (Table 2, Figures 2A–C). There may be two reasons for drought-induced abatement in plant’s photosynthetic rate: one reason is stomatal closure, which prevents the diffusion of CO_2_ to mesophyll cells, further inhibiting CO_2_ assimilation; another is that a decline in carbon assimilation is concomitantly brought about by metabolic impairments (Liu et al., 2017c; Khan et al., 2019). It is believed that PSII-related photochemical reactions are more susceptible to drought than PSΙ, which more effectively reflects the internality of photosynthetic system (Reddy et al., 2004). In our study, regardless of N deposition, drought stress caused a significantly decrease in *F*_v_/*F*_m_ in *F*. *denudata* plants (Figure 2D). Meanwhile, *F*_v_*′*/*F*_m_*′*, Φ_PSII_, and *q*_P_ were also significantly lower in water-stressed plants (Figures 2E–G). This might be due to the fact that PSII down-regulated its original photochemical reactions to weaken the photosynthetic electron transfer rate, as much as possible, to match the reduction in NADPH and ATP requirements in carbon metabolism, thus weakening the oxidative damage caused by the augmentation in excess excitation energy relating to the downregulation of PSII activity (Zhou et al., 2004). However, *F*_v_/*F*_m_ in *F*. *denudata* under drought stress displayed a significant small decline and still remained around 0.77, which showed its photochemical reaction was not severely restricted by drought. Therefore, a noticeable decrease in photosynthetic rate in *F*. *denudata* under drought stress might be mainly attributed to synchronous decreases in *G*_s_ and *C*_i_ (Figures 2B,C), which will further inhibit CO_2_ assimilation capacity (Liu et al., 2017b,c). Evidently, previous studies showed that properly applied nitrogen accelerated the photosynthetic capacity of plants regardless of water availability (Bascuñán-Godoy et al., 2018; Chen et al., 2018; Gao et al., 2018). Our results also confirmed the above finding (Figure 2), which were shown in the following aspects: N deposition increased the number and stacking of chloroplast basal lamellae in well-watered plants as well as ameliorated the ultrastructure of chloroplast in water-stressed plants (Figure 7). Meanwhile, plants under both watering conditions expressed a significant upsurge in chlorophyll contents under N deposition (Table 2), efficiently facilitating absorption, transmission, and conversion of light energy (Cao et al., 2018). Simultaneously, gas exchange parameters were higher in both watering conditions after N deposition (Figures 2A-C). Moreover, N deposition resulted in significantly higher chlorophyll fluorescence parameters in both watering conditions (Figures 2D–G), which indicated that N deposition not only clearly strengthened PSII photochemical reaction of *F*. *denudata* plants in well-watered conditions, but also effectively mitigated the impairment of photosynthetic apparatus (Figure 7) and further alleviated the decline of photosynthetic ability under drought stress (Figure 2).

Being a critical photo-protective mechanism to prevent plant photosynthetic organs from being damaged by photo-oxidative stress under environmental stress, heat dissipation tightly associates to the xanthophyll cycle and is usually measured by NPQ (García-Plazaola et al., 2003; Bascuñán-Godoy et al., 2018). In the present study, regardless of N deposition, NPQ and L content increased in *F*. *denudata* plants as well as DEPS under drought stress (Figure 2H, Table 2), which was helpful to reduce the photo-oxidation damage caused by excess light energy. Conversely, the above parameters of *F*. *denudata* had the opposite trend after N deposition irrespective of water availability (Figure 2H, Table 2), thus N deposition enhanced its carbon assimilation capacity, which were consistent with the elevated *P*_n_ of *F*. *denudata* (Figure 2A). These results were similar to other published researches (Liu et al., 2017b,c).

### Effects of Drought Stress and N Deposition on Active Oxygen Metabolism

Plants respond to water deficit by inducing stomatal closure, greatly restricting CO_2_ diffusion into mesophyll cells, reducing CO_2_ assimilation capacity and, ultimately, causing photochemical and biochemical imbalances in photosynthesis (de Castro et al., 2020). Contemporarily, excess ROS were produced under water stress, causing oxidative damage and affecting proteins, carbohydrates, lipids, and nucleic acids (Gallego et al., 2012). To prevent oxidative damage, plants have evolved a complex antioxidant system including plant-specific ROS-scavenging enzymes and nonenzymatic antioxidants (Ahmad et al., 2016; Fang et al., 2018). In the present study, after drought, the leaves and roots of *F*. *denudata* suffered from oxidative damage (given the higher MDA content) because of the rise of ROS levels in N non-deposition conditions, so did in roots under N deposition conditions, whereas the leaves of *F*. *denudata* showed aggravation of membrane lipid peroxidation only in N deposition conditions (Figures 5, 6). However, some antioxidants and antioxidant enzyme activity levels were observed to be different in leaves and roots of water-stressed plants (Figure 3, Table 3), in order to minimize oxidative damage caused by drought stress. Noticeably, the leaves and roots of *F*. *denudata* differed in active oxygen metabolism in response to drought stress, a result essentially in agreement with previous publications (Du et al., 2017; Agami et al., 2018; Guo et al., 2018; Song et al., 2019). In addition, in the present investigation, it was interesting to notice that in well-watered plants, N deposition despite causing a rise in ROS levels, neither resulted in membrane lipid peroxidation damage, nor significantly changed the antioxidant systems broadly (Figure 3, Table 3). This possibly attributable to the elevated ROS acting as signaling molecules to regulate some physiological metabolism process (Møller et al., 2007; Niu and Liao, 2016). In water-stressed plants, most antioxidant enzyme activities and the contents of antioxidants in leaves were significantly reduced by N deposition; however, the activities of CAT, APX, and DHAR in roots were significantly increased after N deposition (Figure 3, Table 3). These results demonstrated that N deposition alleviated drought-induced oxidative damage of *F*. *denudata* by enhancing the antioxidant defense system in the roots, reducing the accumulation of ROS in leaves, and weakening the induction of the antioxidant defense system in leaves (Figure 3, Table 3). Therefore, there were some differences of the antioxidant system in leaves and roots of water-stressed plants in response to N deposition. Similarly, other studies have found that nitrogen application relieved the effects of drought stress on oxidative damage *via* regulating antioxidant defense system in leaves and roots (Song et al., 2019; Wang et al., 2019).

Effects of drought stress and N deposition on osmotic adjustment.

Plants indeed change carbon and nitrogen metabolisms to combat drought stress, further enhancing its drought adaptability (Bartels and Sunkar, 2005). This happens namely osmotic adjustment, which is a prime drought stress adaptive engine in support of plant production (Blum, 2017). Generally, soluble sugar, soluble protein, and proline are three of the most important osmotic regulating substances (Keunen et al., 2013). Moreover, proline and soluble sugar can also act as antioxidants, which play diverse roles in removing ROS, protecting membrane integrity, protein, and enzyme stability as well as physiological metabolism processes (Ali and Golombek, 2016; Agami et al., 2018). This study found that regardless of N deposition, the leaves and roots displayed higher soluble sugar content under drought stress (Figures 4A,D), indicating that soluble sugar is a central osmoregulation substance; however, the leaves and roots showed lower soluble protein (Figures 4B,E), possibly due to drought stress hindering protein synthesis. These results were similar to those in most previous studies (Agami et al., 2018; Han et al., 2019). Meanwhile, after drought stress, proline in leaves significantly increased irrespective of N deposition, while it only significantly increased in the roots in N deposition plants (Figures 4C,F). Other studies have shown that the response of proline to water deficit was different according to the plant compartments (Maraghni et al., 2014; Azzeme et al., 2016). The possible reason for our results is that drought stress blocked the distribution process of proline synthesized in leaves to the roots under N non-deposition conditions. These results stated clearly that both leaves and roots activated osmotic adjustment mechanisms to reduce the damage caused by drought-induced osmotic imbalance.

In addition, N deposition significantly increased the accumulation of soluble sugar in leaves and soluble protein in leaves and roots under well-watered conditions (Figure 4). One of the reasons for this is the accelerated photosynthesis of plant leaves after N deposition (Figure 2), resulting in more carbon and nitrogen assimilation (Agami et al., 2018). On the other hand, N is indeed positively related to protein synthesis due to it being a necessary component of all proteins (Yin et al., 2012). The different changes of soluble sugar in leaves and roots may be related to the regulation of photoassimilate distribution between source and sink, and the ability of physiological metabolism regulation of plants. However, three osmoregulation substances in leaves and roots of water-stressed plants were evidently increased under N deposition (Figure 4), demonstrating that N deposition plays a vital role in the process of regulating the concentration of osmotic substances to actively reduce osmotic potential under drought stress. Some studies also found that nitrogen application could increase the contents of osmoregulation substance in plants under drought stress, suggesting that osmotic adjustments are the main strategy to improve drought adaptability (Razzaq et al., 2017; Shi et al., 2017).

### Effects of Drought Stress and N Deposition on Ultrastructure

The integrity of plant ultrastructure is the most basic elements for plant physiological metabolism. Being important organelles responsible for photosynthesis and energy flow in plants, chloroplasts and mitochondria are the main objects for ultrastructure observation. It was reported that the ultrastructural damage in leaves and roots with different degrees were observed undergoing drought stress (Das et al., 2015; Han et al., 2019). Our study was accordant with the above result; regardless of N deposition, more serious ultrastructural damage to leaves and roots were found in water-stressed plants than in well-watered plans, but particularly in N non-deposition plants (Figure 7). Additionally, N deposition increased the number and stacking of chloroplast lamellae as well as the number of mitochondria in roots in well-watered plants; moreover, it also mitigated the damage to chloroplasts and mitochondria under drought stress (Figure 7), improving the function of the two major organelles and further enhancing the adaptation of *F*. *denudata* under drought stress. Our results well-supported the claim that proper N application helps to maintain ultrastructure (Yin et al., 2012; Liu et al., 2018).

## Conclusion

This study provides new evidence that N deposition alleviates the negative effects of drought on plants. It can be concluded that nitrogen deposition increased heat dissipation in leaves, the antioxidant capacity in roots as well as osmotic adjustment in leaves and roots to alleviate oxidative damage as well as improve photosynthetic and growth performance in *F. denudate* under drought stress. Therefore, N deposition distinctly improved drought resistance of *F. denudate* through promoting the co-operation of different protection mechanisms in leaves and roots.

## Data Availability Statement

The raw data supporting the conclusions of this article will be made available by the authors, without undue reservation.

## Author Contributions

YW designed the practical part of the study, SW and TR analyzed the data, and SW and JT drafted the manuscript. All authors contributed to the article and approved the submitted version.

## Funding

This work was supported by the Second Tibetan Plateau Scientific Expedition and Research Program (STEP; grant no. 2019QZKK0303).

## Conflict of Interest

The authors declare that the research was conducted in the absence of any commercial or financial relationships that could be construed as a potential conflict of interest.

## Publisher’s Note

All claims expressed in this article are solely those of the authors and do not necessarily represent those of their affiliated organizations, or those of the publisher, the editors and the reviewers. Any product that may be evaluated in this article, or claim that may be made by its manufacturer, is not guaranteed or endorsed by the publisher.
